# Pediatric blunt abdominal trauma with horizontal duodenal injury in school baseball: A case report

**DOI:** 10.1097/MD.0000000000024089

**Published:** 2021-01-15

**Authors:** Teppei Tokumaru, Ryozo Eifuku, Kenichi Sai, Hideaki Kurata, Michiaki Hata, Joji Tomioka

**Affiliations:** Department of Acute Medicine and Surgery, Yonemori Hospital, 1-7-1, Yojiro, Kagoshima-City, Kagoshima, 890-0062, Japan.

**Keywords:** abdominal trauma, duodenal injury, duodenum, pancreatic injury, pediatric surgery, sport injury

## Abstract

**Rationale:**

Pediatric sports injuries, including those from baseball, most often are musculoskeletal injuries and rarely include blunt abdominal injuries. Duodenal injury is rare and often associated with other organ injuries. Because it has a relatively high mortality, early recognition and timely treatment are needed. Here, we report a case of successful treatment of a pediatric patient with duodenal injury incurred in the context of school baseball.

**Patient concerns:**

A 13-year-old boy suffered blunt abdominal trauma and a right-hand injury caused by beating his abdomen strongly with his own right knuckle after he performed a diving catch during a baseball game. On the following day, the abdominal pain had worsened.

**Diagnoses:**

Computed tomography led to a suspicion of injury to the horizontal part of the duodenum.

**Interventions:**

The duodenal injuries were repaired by simple closure. On the 10th post-operative day, an abscess formed in the retroperitoneal cavity because of an occult pancreatic injury. Ultrasound-guided percutaneous drainage of the cavity was performed.

**Outcomes:**

The post-operative course of the abscess drainage was uneventful. The patient was discharged from our hospital on day 72 after admission and was in good health at the 9-month follow-up.

**Lessons:**

Regardless of the type of injury, we must assess the life-threatening conditions that can be expected based on the mechanism of the injury. In duodenal injuries, it is critical to perform surgical procedures and post-operative management based on the assumption of injuries to other organs.

## 1 Introduction

Pediatric sports injuries, including those from baseball, include mainly musculoskeletal injuries and rarely blunt abdominal injuries.^[1]^ Pediatric blunt abdominal injuries are mainly caused by traffic accidents and falls, and few cases have been reported in relation to sports injuries.^[2]^ Duodenal injury is rare among abdominal injuries and accounts for 0.2% to 3.7% of all trauma-related laparotomies. Isolated duodenal injury is rare, as it is often associated with 1 to 4 other abdominal organ injuries.^[3]^ Because it has a relatively high mortality (6–25%), early recognition and timely treatment are imperative and decrease mortality.^[4,5]^ In the initial trauma assessment, it is important to avoid underestimating the trauma regarding life-threatening injury based on the details of the mechanism of injury, regardless of its type. Here, we report a case of successful treatment of a pediatric patient with severe abdominal injury incurred in the context of school baseball.

## 2 Case presentation

**Figure 1:**
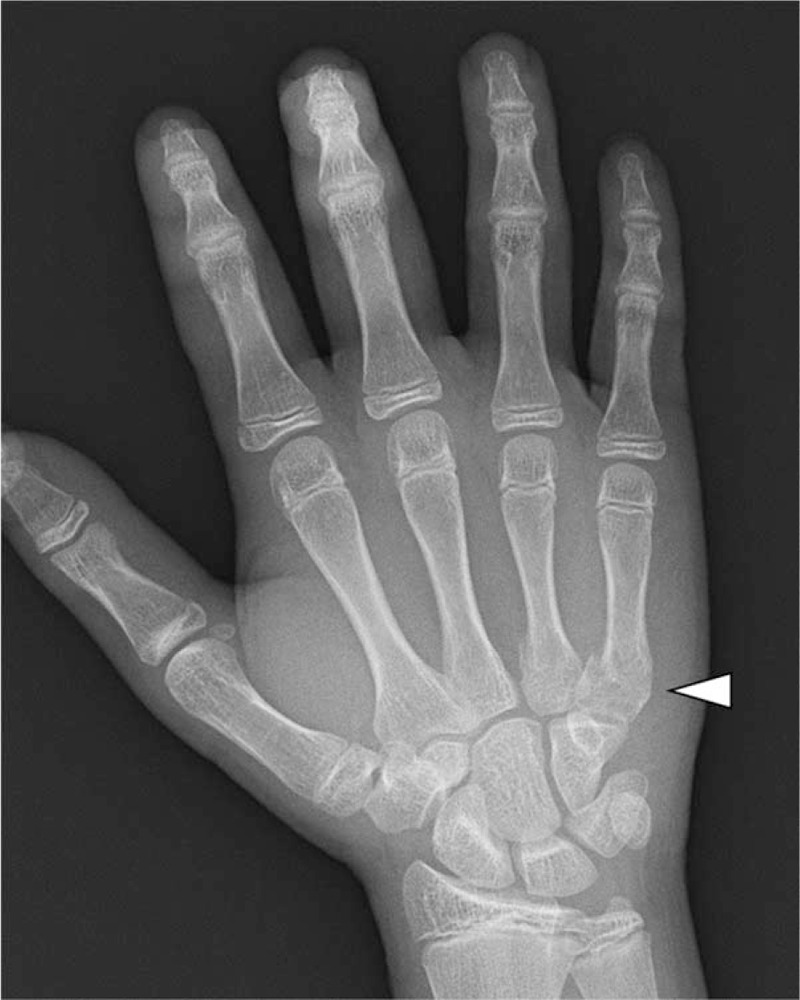
Pre-hospital X-ray: a right 4th and 5th metacarpal fracture called a boxer's fracture (white arrowhead) was noted.

A 13-year-old boy who was previously healthy with no medical history suffered blunt abdominal trauma and right-hand injury caused by beating his abdomen strongly with his own right knuckle after he performed a diving catch during a school baseball game. The patient visited a local hospital on the same day with abdominal and right-hand pain. Abdominal ultrasonography revealed no significant findings, and the patient returned home with a diagnosis of a right 4th and 5th metacarpal fracture (called a boxer's fracture) (Fig. 1). On the following day, the abdominal pain worsened, and the patient revisited the local hospital. At that time, abdominal ultrasonography revealed liquid retention around the kidney. Therefore, the patient was transferred to our hospital via helicopter transportation across the prefectural border.

**Figure 2:**
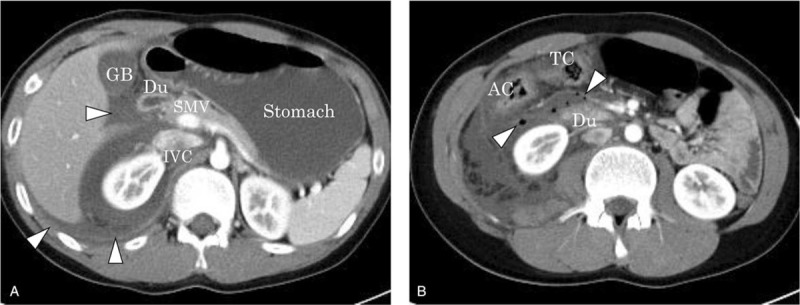
Pre-hospital computed tomography findings. (A) Image depicting fluid retention and dilatation of the stomach. Fluid retention around the hepatorenal recess space, also referred to as the pouch of Morison, was revealed (white arrowheads). Du = duodenum, GB = gallbladder, IVC = inferior vena cava, SMV = superior mesenteric vein. (B) Image depicting the accumulation of luminal gas within the posterior peritoneal cavity around the duodenum (white arrowheads). AC = ascending colon, TC = transverse colon.

**Figure 3:**
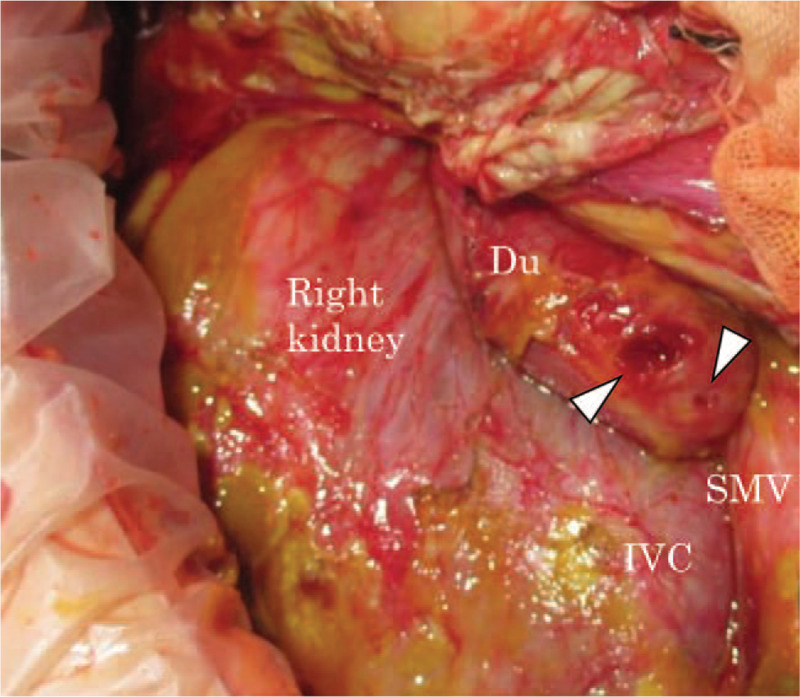
Intra-operative findings: the colon laceration showed a serosal muscular layer injury of about 10 cm along the long axis, with a Grade I American Association for the Surgery of Trauma (AAST) injury score.

**Figure 4:**
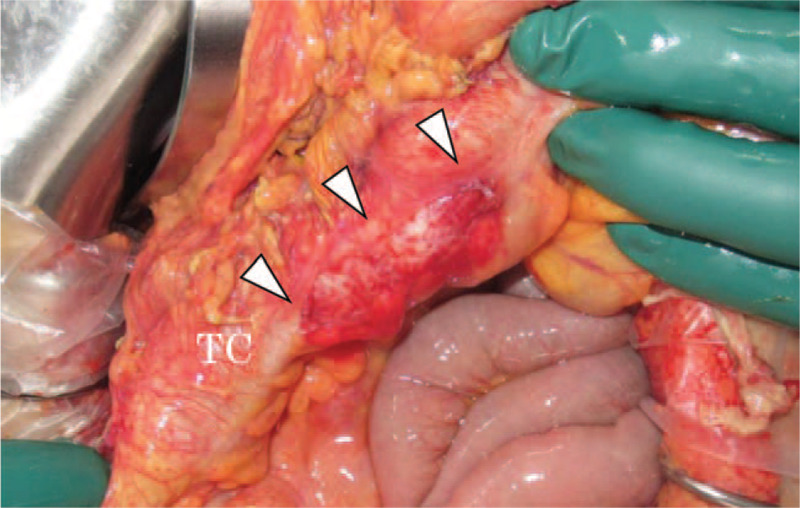
Intra-operative findings after the execution of a Kocher maneuver. Two duodenal injuries (perforations) were observed on the horizontal part of the duodenum (white arrowheads). This was scored a Grade II injury, as per the organ injury scoring table of the American Association for the Surgery of Trauma. Du = duodenum, IVC = inferior vena cava, SMV = superior mesenteric vein.

Physical examination revealed a body temperature of 37.1°C, a blood pressure of 117/98 mm Hg, a heart rate of 142 bpm, a respiratory rate of 36 times/min, and a consciousness level of alert, but slightly restless. His general appearance was sick with perspiration. His abdomen showed distension and hard tenderness. A blood test revealed a hemoglobin level of 15.3 g/dL (normal range, 11.0–15.1 g/dL), a white blood cell count of 9200/μL (normal range, 3500/μL–9300/μL), and a platelet count of 31,800/μL (normal range, 13,000/μL–36,000/μL). The results of laboratory investigations showed a C-reactive protein level of 13.81 mg/dL (normal range, 0.00–0.30 mg/dL) and an amylase level of 394 U/L (normal range, 40–125 U/L). Abdominal computed tomography (CT) indicated ascites retention with extra-luminal gas from the retroperitoneal to the peritoneal cavity, suggesting intestinal injury (Fig. 2); therefore, we decided to perform emergency surgery. At this point, 21 h had passed after the injury. The laparotomy findings included bloody and digestive-juice ascites (1000 mL) around the liver, spleen, and bladder. The surgical examination of the ascending colon via a Kocher maneuver revealed an injury of the transverse colon and the horizontal part of the duodenum, whereas no pancreatic injury was observed upon macroscopic examination. The colon laceration included a serosal muscular layer injury of about 10 cm along the long axis, with a Grade I American Association for the Surgery of Trauma (AAST) injury score (Fig. 3). The duodenal laceration showed 2 perforations of 1 and 2 cm, respectively, with a Grade II AAST injury score (Fig. 4). The duodenal injuries were simply repaired by merging 2 perforations into 1 via full-thickness incision plasty. The injury of the transverse colon was repaired by suturing the serosal muscle layer.

On the 10th post-operative day, he showed no abdominal symptom, but presented with fever. A blood test showed a white blood cell count of 20,700/μL, a C-reactive protein level of 8.26 mg/dL, and an amylase level of 226 U/L. A CT scan showed fluid retention on the retroperitoneum cavity; thus, percutaneous drainage under ultrasound guidance was performed. Radiographic imaging from the drainage catheter revealed no duodenal or pancreatic fistula. Radiographic imaging of the duodenum revealed no anastomotic leakage. A test of the drainage fluid revealed an amylase level of 1384 U/L and the presence of methicillin-sensitive *Staphylococcus aureus* (MSSA). These findings are indicative of an occult pancreatic injury. The post-drainage course was uneventful and the drainage catheter was removed after the dead space was closed, with no infection noted on the 34th hospital day. The patient was discharged from our hospital 72 days after admission and is in good health 5 months after the injury. The consent of the patient and parents regarding the publication of the paper was obtained. This study was approved by our institutional ethics committee for pediatric cases.

## 3 Discussion

Pediatric sports injuries, including ones that occur while playing baseball, often result in musculoskeletal injuries.^[2]^ However, they can result in abdominal trauma, such as duodenal injury, which is a life-threatening injury. The mechanism for duodenal injury of blunt abdominal trauma involves crushing, compression, and traction injury. The blunt abdominal trauma can result in the crushing of the duodenum between an external hard object and the spine. Furthermore, the impact of abrupt acceleration or deceleration at the point of duodenal attachment at the ligament of Treitz can result in an increase in the intraluminal pressure and traction of the duodenum.^[3,5]^ Duodenal injuries are rare, and their anatomical diagnosis, especially in the horizontal part, is often delayed; moreover, these injuries can be accompanied by injuries to other organs.^[6]^

In the case reported here, the dominant right hand of the pediatric patient struck his abdomen when he dove to catch a ball while playing baseball. The initial diagnosis was right-hand fracture, a so-called “boxer's fracture,” alone. However, the patient's abdominal discomfort worsened, and he revisited a hospital on the day following the injury. Pediatric abdominal injuries are caused mainly by traffic accidents and falls, while sports-related abdominal injuries are uncommon. Moreover, most sports injuries are musculoskeletal injuries alone.^[2]^ Our search of the medical literature using PubMed found no reports of abdominal injuries incurred while playing baseball. In the present case, the abdominal injury was caused by an amount of energy that was large enough to break the patient's fist on impact with his abdomen while diving to catch a ball. One factor that likely contributed to this injury was the fact that, anatomically, children have less abdominal fat. In addition, the patient exhibited a shock status with chills upon arrival at the hospital, although his blood pressure was within the normal range. Therefore, such a presentation must not be underestimated, as physiological signs in children are different from those observed in adults.^[7,8]^ In sports injuries, we must consider the life-threatening status that can be expected based on the mechanism of the injury, regardless of its type.

In the present case, the injury was identified in the horizontal part of the duodenum with an abscess from the peritoneal to the intraperitoneal cavity. Considering the risk of post-operative complications, such as anastomotic leakage and stenosis, bypass surgery and gastrointestinal decompression using drainage, in addition to simple closure, have been reported for duodenal injuries.^[5]^ However, there is no consensus regarding the standard surgical procedure for duodenal injuries, as they need to be determined comprehensively based on physical condition, morphology, site, and time since the onset of the injury.^[9]^ Therefore, to avoid overlooking the horizontal part of the duodenum because of its location in the retroperitoneal cavity, we performed a Kocher maneuver adequately to identify not only the main trunk of the superior mesenteric vessels, but also the overall horizontal part of the duodenum.^[10]^ We found 2 adjoining areas of duodenal injury and we determined that sufficient debridement would be possible with adequate blood flow via a single plastic port. Thus, the duodenal injuries were simply repaired by merging 2 perforations into 1 via full-thickness incision plasty. On the 10th post-operative day, an abscess formed in the retroperitoneal cavity and percutaneous drainage was performed under ultrasound guidance. Furthermore, contrast-enhanced imaging of the drainage of the duodenum and pancreatic duct did not indicate the presence of fistula, and oral gastrographic imaging showed no anastomotic leakage of the duodenal repair. Moreover, high levels of drainage amylase and detection of MSSA suggested that the infection and abscess formation were caused by a pancreatic fistula stemming from an occult pancreatic injury that was not evident on pre-operative imaging and intra-operative findings.^[11]^ In addition, the pancreatic tissue may have been severely damaged by the digestive juice over the 21 h that elapsed after the injury. The post-operative drainage was clear, and the drainage tube was removed on the 4th post-operative day. However, early detection might have been possible if the drainage amylase levels had been measured prior to its removal. In cases of injury of the horizontal part of the duodenum, it is advisable to assume occult pancreatic injury, even if it is not evident from pre-operative images or intra-operative findings.^[11]^

In conclusion, we report a case of a pediatric abdominal blunt trauma during a baseball game that caused a duodenal injury. Regardless of the background of the injury, we must consider the life-threatening conditions that can be expected based on the mechanism of the injury. Pediatric injuries must not be underestimated because children are anatomically and physiologically different from adults. In duodenal injuries, it is critical to perform surgical procedures and post-operative management based on the assumption of injuries to other organs. We believe that this is relevant for treatment strategies for all abdominal trauma.

## Author contributions

**Conceptualization:** Teppei Tokumaru.

**Investigation:** Eifuku Ryozo.

**Methodology:** Joji Tomioka.

**Resources:** Kenichi Sai, Hideaki Kurata.

**Validation:** Michiaki Hata, Joji Tomioka.

**Writing – original draft:** Teppei Tokumaru.

**Writing – review & editing:** Teppei Tokumaru, Joji Tomioka.

## Abbreviations

Abbreviations: AAST = American Association for the Surgery of Trauma, CT = computed tomography, MSSA = methicillin-sensitive *Staphylococcus aureus*.
